# Multi-type feature fusion based on graph neural network for drug-drug interaction prediction

**DOI:** 10.1186/s12859-022-04763-2

**Published:** 2022-06-10

**Authors:** Changxiang He, Yuru Liu, Hao Li, Hui Zhang, Yaping Mao, Xiaofei Qin, Lele Liu, Xuedian Zhang

**Affiliations:** 1 College of Science, University of Shanghai for Science and Technology, Shanghai, 200093 China; 2 School of Optical-Electrical and Computer Engineering, University of Shanghai for Science and Technology, Shanghai, 200093 China; 3 Institute of Interdisciplinary Integrative Medicine Research, Shanghai University of Traditional Chinese Medicine, Shanghai, 201203 China; 4 School of Mathematics and Statistis, Qinghai Normal University, Xining, 810008 China

**Keywords:** Multi-type feature fusion, Graph neural network, Gating mechanism, Link prediction

## Abstract

**Background:**

Drug-Drug interactions (DDIs) are a challenging problem in drug research. Drug combination therapy is an effective solution to treat diseases, but it can also cause serious side effects. Therefore, DDIs prediction is critical in pharmacology. Recently, researchers have been using deep learning techniques to predict DDIs. However, these methods only consider single information of the drug and have shortcomings in robustness and scalability.

**Results:**

In this paper, we propose a multi-type feature fusion based on graph neural network model (MFFGNN) for DDI prediction, which can effectively fuse the topological information in molecular graphs, the interaction information between drugs and the local chemical context in SMILES sequences. In MFFGNN, to fully learn the topological information of drugs, we propose a novel feature extraction module to capture the global features for the molecular graph and the local features for each atom of the molecular graph. In addition, in the multi-type feature fusion module, we use the gating mechanism in each graph convolution layer to solve the over-smoothing problem during information delivery. We perform extensive experiments on multiple real datasets. The results show that MFFGNN outperforms some state-of-the-art models for DDI prediction. Moreover, the cross-dataset experiment results further show that MFFGNN has good generalization performance.

**Conclusions:**

Our proposed model can efficiently integrate the information from SMILES sequences, molecular graphs and drug-drug interaction networks. We find that a multi-type feature fusion model can accurately predict DDIs. It may contribute to discovering novel DDIs.

## Introduction

Drug-Drug interactions (DDIs) refer to the presence of one drug changing the pharmacological activity of another, which may produce some side effects and even injury or death. At the same time, multiple drug combinations to treat diseases are inevitable. So, it is crucial to predict potential DDI. Traditional methods of DDI prediction depend on in vivo and in vitro experiments. However, due to its limited environment, too small scale, cumbersome and expensive process, the ability to predicting DDI is greatly limited. Therefore, an efficient computational method is needed to predict DDI.

In the past several years, people have proposed methods based on machine learning [1–4] to solve this problem. Qiu et al. [5] summarized some methods based on machine learning. Deng et al. [6] used chemical structure to learn the representation of DDIs in representation module, and then predicted some rare events with few examples in comparing module. Deng et al. [7] predicted DDI using different drug features and constructed deep neural networks (DNN). Zhang et al. [8] predicted DDI using manifold regularization.

Recently, graph-based representation learning has been applied to Drug-Drug interaction. Drugs are compounds, each of which can be represented by a molecular graph with the atom as the node and the chemical bond as the edge, or a Simplified Molecular Input Line Entry System (SMILES) sequence. In Drug-Drug interaction networks, by treating the drug as the node and the interaction as the edge, DDI prediction can be regarded as link prediction tasks. Graph neural network (GNN) has made some progress in DDI prediction [9–13]. Feng et al. [14] predicted DDI using Graph Convolutional Network (GCN) and DNN. In addition, there are also many methods about multi-type DDI prediction [15–17]. Nyamabo et al. [18] proposed to predict DDIs by the interactions between drug substructures. Then, Nyamabo et al. [19] used gating devices to learn the chemical substructures of drugs. Chen et al. [20] used the bi-level cross strategy to fuse the structural information and knowledge graph information of drugs.

Although the models mentioned have achieved significant results, there are still some limitations: (i) The models mentioned are generally limited to only considering the structure, sequence or interaction information of the drugs, without considering the synergistic effects between them. (ii) For molecular graphs, only applying GNN can extract the local features for the atoms of the molecular graph, but it is difficult to propagate the information in the graph remotely to capture the global features for the molecular graph. (iii) In drug-drug interaction networks, node features obtained by stacking multi-layer GNNs will be smoothed and blurred, which loses the diversity of node features.

To address above issues, this paper proposes an end-to-end learning framework for DDI prediction, namely MFFGNN. In MFFGNN, we first utilize deep neural networks to capture the intra-drug features from SMILES sequences and molecular graphs. For SMILES sequences, MFFGNN applies the bi-directional gate recurrent unit neural network [21] to extract local chemical context information from the sequences. For molecular graphs, MFFGNN not only utilizes graph interaction networks [22] but also graph warp unit [23] to extract both the global features for the molecular graph and the local features for each atom of the molecular graph. In addition, MFFGNN takes the intra-drug features as the initial features of the nodes in the DDI network and uses GCN encoder to fuse the intra-drug features and external DDI features to update the drug representation. Finally, we predict the missing interactions in the DDI graph through Multi-layer Perceptron (MLP).

Overall, the main contributions of this paper are summarized as follows:

- We propose a novel model MFFGNN for DDI prediction, which fuses the topological information in molecular graphs, the interaction information between drugs and the local chemical context in SMILES sequences.
- To better learn the topological structure of drugs, we propose a molecular graph feature extraction module (MGFEM) to extract the global features for the molecular graph and the local features for each atom of the molecular graph.
- We conduct extensive experiments on three real datasets with different scales to demonstrate the superiority of our model.

## Related works

### Drug-drug prediction

Drug-Drug prediction has always been a worthy research direction in pharmacology. Most of previous work depended on in vivo and in vitro experiments. However, they do not scale well due to the limitations of the laboratory environment [24]. Subsequently, machine learning has been proposed to solve this problem. Similarity-based methods calculated specific similarity measures [25–29], e.g., drug structure, targets, side effects, genomic properties, therapeutic, etc., while combined with machine learning models for drug prediction. Ryu et al. [30] predicted the type of drug-drug interactions using DNN based on the similarity of the chemical structure of drugs. Graph-based methods predicted drug-drug interactions by learning the molecular graph [31] or interaction graph [32]. Shang et al. [33] modeled drugs as nodes and DDI as links, so tasks as link prediction problems.

### Graph neural network

Recently, as a neural network method on graph domain, the study of graph neural network (GNN) has received great attention. With the development of GNN, many variants based on GNN came out one after another [34–36]. Rahimi et al. [37] proposed to control the transmission of neighbourhood information through gating operation. With the increasing popularity of GNN, researchers are using GNN models for DDIs [38]. For example, Duvenaud et al. [39] used GNN to perform molecular modeling by extracting molecular circular fingerprints. Lin et al. [40] used knowledge graph neural network (KGNN) to mine their associated relations in knowledge graph to solve the DDI prediction problem. Bai et al. [41] proposed to learn drug feature representation by a Bi-level Graph Neural Network (BI-GNN) to solve biological link prediction tasks. MIRACLE [42] is most relevant to our work.

## Methods

### Preliminaries

We define the drug set as $D\!\!=\!\!\{d_{1}, \ldots , d_{n}\}$ and its corresponding SMILES sequence set as $Q=\left\{ q_{1}, q_{2}, \ldots , q_{n}\right\}$, where *n* represents the number of drugs. We define the molecular graph as ${\mathbb {G}}=({\mathbb {V}}, {\mathbb {E}})$, where ${\mathbb {V}}$ and ${\mathbb {E}}$ represent the sets of atoms and chemical bonds, respectively, and interaction graph as ${\mathcal {G}}=({\mathbb {G}}, {\mathbb {L}})$, where ${\mathbb {L}}$ represents the links between drugs. We use $d_{h}$ to define the dimension of the representation of the atom and chemical bond and $d_{g}$ to define the dimension of the representation of the drug.

*Problem description* The DDI prediction problem is regarded as the link prediction task on the graph. The interaction graph ${\mathcal {N}}$ can be represented by an adjacency matrix ${\mathbf {A}} \in {\mathbb {R}}^{n \times n}$ with each element $a_{i j} \in \{0,1\}$. Given two drug nodes, the DDI prediction problem is defined to predict whether there is an interaction between them.

### Overview of MFFGNN

**Fig. 1:**
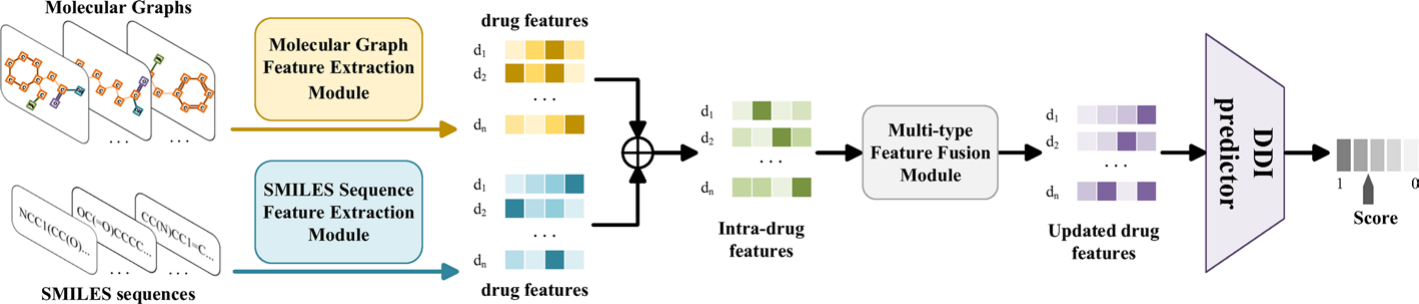
Overview of MFFGNN, where $\bigoplus$ is sum. The MFFGNN uses SMILES sequences and molecular graphs as inputs to the model, and then extracts the intra-drug features through the MGFEM and SSFEM modules, respectively. Then, MFFGNN fuses the intra-drug features and external DDI features through MFFM module to obtain the updated drug features. Finally, the final predicted value is obtained by DDI predictor

The framework of MFFGNN is shown in Fig. 1, which is divided into the following four modules. In Molecular Graph Feature Extraction Module (MGFEM), we use the graph interaction network with graph wrap unit to extract the topological structure features of the drug from a given molecular graph. In SMILES Sequence Feature Extraction Module (SSFEM), we employ the bi-directional gate recurrent unit to extract local chemical context from a given SMILES sequence. In Multi-type Feature Fusion Module (MFFM), we apply GCN encoder to fuse the intra-drug features and external DDI features to update the drug representation. Finally, we predict the missing interactions in the DDI graph through MLP.

### Molecular graph feature extraction module

**Fig. 2:**
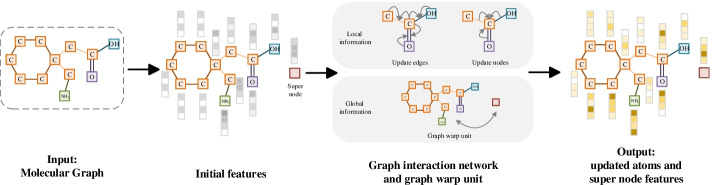
Overview of MGFEM. The MGFEM module applies graph interaction network and graph wrap unit to extract local information and global information of the molecular graph. When extracting the local information, the module updates the edge feature before updating the node feature. When extracting the global information, the module utilizes a supernode to promote the global propagation of information

The Molecular Graph Feature Extraction Module (MGFEM) is shown in Fig. 2. Molecular graphs are an important expression for drugs. We use RDKit [43] tool to construct the molecular graph ${\mathbb {G}}$ based on SMILES sequence. First, we obtain the initial features ${\mathbf {v}}_{i}^{(in)}$ of each atom according to atom symbol, formal charge, whether the atom is aromatic, its hybridization, chirality, etc. Similarly, we obtain the initial features ${\mathbf {e}}_{ij}^{(in)}$ of each bond according on the type of bond, whether the bond is in a ring, whether it is conjugated, etc. Then, the initial atom and chemical bond features are transformed to ${\mathbb {R}}^{d_{h}}$ through a layer neural network, and the calculation process is as follows:

1 $$\begin{aligned} {\mathbf {v}}_{i}^{(0)}&= {\text {ReLU}}({\mathbf {W}}_{v}^{(0)} {\mathbf {v}}_{i}^{(in)}) \end{aligned}$$

2 $$\begin{aligned} {\mathbf {e}}_{ij}^{(0)}&={\text {ReLU}}({\mathbf {W}}_{e}^{(0)} {\mathbf {e}}_{ij}^{(in)}) \end{aligned}$$

where ${\text {ReLU}}$ is the activation function, ${\mathbf {W}}_{v}^{(0)}$ and ${\mathbf {W}}_{e}^{(0)}$ are the learnable weight matrices. To fully extract atom and chemical bond features, we apply graph interaction networks [22]. In graph interaction network, firstly, the features of edge $e_{ij}$ are updated according to the features of its connected nodes and itself, and the process is as follows:

3 $$\begin{aligned} {\mathbf {e}}_{i j}^{(l+1)}={\text {ReLU}}[({\mathbf {e}}_{i j}^{(l)} || {\mathbf {v}}_{i}^{(l)} || {\mathbf {v}}_{j}^{(l)}) {\mathbf {W}}_{e}^{(l)}+{\mathbf {b}}_{e}^{(l)}] \end{aligned}$$

where || is concatenation operation, ${\mathbf {W}}_{e}^{(l)}$ and ${\mathbf {b}}_{e}^{(l)}$ are the learnable weight matrix and the bias of the edge update, respectively. Then, the node features are updated according to the features of its connected edges and itself, and the calculation process is as follows:

4 $$\begin{aligned} {\tilde{{\mathbf {v}}}}_{i}^{(l+1)}\!\!=\!{\text {ReLU}}[({\mathbf {v}}_{i}^{(l)} || \sum _{j \in N(i)} \mathrm {e}_{i j}^{(l+1)}) {\mathbf {W}}_{v}^{(l)}+{\mathbf {b}}_{v}^{(l)}] \end{aligned}$$

where *N*(*i*) represents the neighbor of node *i*.

The above processes can only spread the features of atoms and chemical bonds locally, but cannot spread information globally. Therefore, we propose to extract the global features of the molecular graph by applying graph warp unit (GWU) [23]. The properties of the whole drug often influence drug-drug interaction prediction. The GWU consists of three parts: supernode, transmitter and warp gate.

Supernode: We add a supernode to the graph, which can connect every atom in the molecular graph. Then, the sum of all atom features is taken as the initial feature of the supernode, ${\mathbf {g}}^{(0)}\in {\mathbb {R}}^{d_{h}}$, that is:

5 $$\begin{aligned} {\mathbf {g}}^{(0)}=\sum _{i \in {\mathbb {V}} } {\mathbf {v}}_{i}^{(0)}. \end{aligned}$$

Then, the features of the supernode are updated by a single-layer neural network:

6 $$\begin{aligned} {\tilde{{\mathbf {g}}}}^{(l)} = \text {tanh} ({\mathbf {W}}_{g}^{(l)} {\mathbf {g}}^{(l-1)}) \end{aligned}$$

where ${\mathbf {W}}_{g}^{(l)}$ are the learnable weight matrix.

Transmitter: The transmitter part gathers information from the atoms and the supernode. Before propagating the atom features to the supernode, we need to transform the form of the information. Different atom features have different degrees of importance relative to the global features. Therefore, the transmitter part applies the multi-head attention mechanism to aggregate different atom features. The calculation process is as follows:

7 $$\begin{aligned} {\mathbf {v}}_{v \rightarrow s}^{(l)}&=\tanh ({\mathbf {W}}_{v \rightarrow s}^{(l)} [{||}_{k=1}^{K} \sum _{i \in {\mathbb {V}} } \alpha _{v, i}^{(k, l)} {\mathbf {v}}_{i}^{(l-1)}]) \end{aligned}$$

8 $$\begin{aligned} \alpha _{v, i}^{(k, l)}&={\text {softmax}}({\mathbf {W}}_{a}^{(k, l)} {\mathbf {o}}_{v, i}^{(k, l)} ) \end{aligned}$$

9 $$\begin{aligned} {\mathbf {o}}_{v, i}^{(k, l)}&= \tanh ({\mathbf {W}}_{a1}^{(k, l)} {\mathbf {v}}_{i}^{(l-1)}) \odot \tanh ({\mathbf {W}}_{a2}^{(k, l)} {\mathbf {g}}^{(l-1)}) \end{aligned}$$

where ${\mathbf {v}}_{v \rightarrow s}^{(l)}$ represents the information propagated from each atom to the supernode at the $l^{th}$ layer, $\alpha _{v, i}^{(k, l)}$ represents the significance score of node *i* at the $k^{th}$ head and the $l^{th}$ layer, $\odot$ represents the product of the elements and $k =1,2, \ldots , K$, *K* represents the number of heads. The information propagated from the supernode to each atom is calculated by the following formula:

10 $$\begin{aligned} {\mathbf {g}}_{s \rightarrow v}^{(l)}=\tanh \left( {\mathbf {W}}_{s \rightarrow v}^{(l)} {\mathbf {g}}^{(l-1)}\right) \end{aligned}$$

where ${\mathbf {g}}_{s \rightarrow v}^{(l)}$ represents the information propagated from the supernode to each atom at the $l^{th}$ layer.

Warp Gate: The warp gate combines the transmitted information and sets the gating coefficients to control the fusion of information. For each atom, gated interpolation is used to fuse the information from the supernode ${\mathbf {g}}_{s \rightarrow v}^{(l)}$ with the updated atom features ${\mathbf {v}}_{i}^{(l)}$:

11 $$\begin{aligned} {\mathbf {v}}_{s \rightarrow i}^{(l)}&=\left( {\mathbf {1}}-\alpha _{s \rightarrow i}^{(l)}\right) \odot \tilde{{\mathbf {v}}}_{i}^{(l)}+\alpha _{s \rightarrow i}^{(l)} \odot {\mathbf {g}}_{s \rightarrow v}^{(l)} \end{aligned}$$

12 $$\begin{aligned} \alpha _{s \rightarrow i}^{(l)}&=\sigma \left( {\mathbf {W}}_{b1 }^{(l)} \tilde{{\mathbf {v}}}_{i}^{(l)}+{\mathbf {W}}_{b2 }^{(l)} {\mathbf {g}}_{s \rightarrow v}^{(l)}\right) \end{aligned}$$

where $\alpha _{s \rightarrow i}^{(l)}$ represents the gating coefficient during the transmission from supernode to each atom and ${\mathbf {v}}_{s \rightarrow i}^{(l)}$ represents the information transmitted to each atom. For supernode, gated interpolation is used to fuse information from atoms ${\mathbf {v}}_{v \rightarrow s}^{(l)}$ with updated supernode features $\tilde{{\mathbf {g}}}^{(l)}$:

13 $$\begin{aligned} {\mathbf {g}}_{i \rightarrow s}^{(l)}&=\left( {\mathbf {1}}-\alpha _{s \rightarrow i}^{(l)}\right) \odot \tilde{{\mathbf {g}}}^{(l)}+\alpha _{s \rightarrow i}^{(l)} \odot {\mathbf {v}}_{v \rightarrow s}^{(l)} \end{aligned}$$

14 $$\begin{aligned} \alpha _{i \rightarrow s}^{(l)}&=\sigma \left( {\mathbf {W}}_{s1 }^{(l)} \tilde{{\mathbf {g}}}^{(l)}+{\mathbf {W}}_{s2 }^{(l)} {\mathbf {v}}_{v \rightarrow s}^{(l)}\right) \end{aligned}$$

where $\alpha _{i \rightarrow s}^{(l)}$ represents the gating coefficient during the transmission from atom to supernode and ${\mathbf {g}}_{i \rightarrow s}^{(l)}$ represents the information transmitted to supernode. Finally, the updated features of each atom and supernode are calculated through the gated recurrent units (GRU) [44]:

15 $$\begin{aligned} {\mathbf {v}}_{i}^{(l)}&={\text {GRU}}_{v}\left( {\mathbf {v}}_{i}^{(l-1)}, {\mathbf {v}}_{s \rightarrow i}^{(l)}\right) \end{aligned}$$

16 $$\begin{aligned} {\mathbf {g}}^{(l)}&={\text {GRU}}_{g}\left( {\mathbf {g}}^{(l-1)}, {\mathbf {g}}_{i \rightarrow s}^{(l)}\right) . \end{aligned}$$

By applying this module to the whole dataset, we obtain the feature matrix ${\mathbf {G}}\in {\mathbb {R}}^{n \times d_{g}}$ based on the molecular graph.

### SMILES sequence feature extraction module

**Fig. 3:**
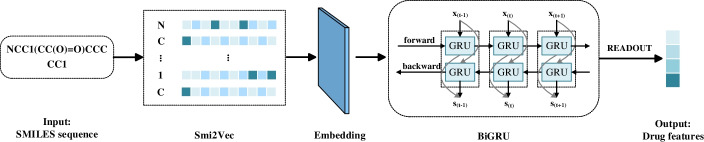
Overview of SSFEM. The SSFEM module applies Smi2Vec and BiGRU to extract features from SMILES sequences. Then, the whole drug features are obtained through the readout layer

Drugs are commonly represented by the SMILES sequences, which are composed of molecular symbols. SMILES sequences also contain rich features compared with molecular graphs. The molecular graphs of the drug provide how the atoms are connected, while the SMILES sequences provide the functional information of the atoms and long-term dependency representations. To capture the local chemical context in SMILES sequences, we first utilized the embedding method to construct an atomic embedding matrix, and then input it into the Bi-directional Gate Recurrent Unit (BiGRU) neural network to obtain the entire drug representation. SMILES Sequence Feature Extraction Module (SSFEM) is shown in Fig. 3.

Nowadays, most methods encode SMILES sequence by label or one-hot encoding. However, one-hot encoding and label ignore the context information of the atom. Therefore, to explore the function of the atom in the context, we propose to encode SMILES sequences by an advanced embedding method, *Smi2Vec* [45]. Specifically, for SMILES sequences $q_{1}$, we divide them into a series of atomic symbols by space. Then, we map each atom to an embedding vector according to the pre-trained embedding dictionary. Finally, we aggregate the embedding vectors of atoms to obtain an embedding matrix ${\mathbf {X}} \in {\mathbb {R}} ^{{m} \times d_{h}}$, in which *m* is the number of atoms and each row is the embedding of an atom.

We apply a layer of BiGRU [21] on the embedding matrix ${\mathbf {X}}$. BiGRU trains the input data with two GRUs in opposite directions, as shown in Fig. 3. The current hidden state of BiGRU can be described as follows: $\overrightarrow{{\mathbf {s}}_{t}}\!\!=\!\!{\text {GRU}}({\mathbf {x}}_{t}, \overrightarrow{{\mathbf {s}}_{t-1}})$ and $\overleftarrow{{\mathbf {s}}_{t}}\!\!=\!\!{\text {GRU}}({\mathbf {x}}_{t}, \overleftarrow{{\mathbf {s}}_{t-1}})$ , where ${\text {GRU}}(\cdot )$ represents a non-linear transformation of the input vector. Therefore, the hidden state ${\mathbf {s}}_{t}$ at time *t* can be expressed by the weighted sum of $\overrightarrow{{\mathbf {s}}_{t}}$ and $\overleftarrow{{\mathbf {s}}_{t}}$, which is expressed as follows:

17 $$\begin{aligned} {\mathbf {s}}_{t}={\mathbf {W}}_{t} \overrightarrow{{\mathbf {s}}_{t}}+{\mathbf {V}}_{t} \overleftarrow{{\mathbf {s}}_{t}}+{\mathbf {b}}_{t} \end{aligned}$$

where ${\mathbf {W}}_{t}$ and ${\mathbf {V}}_{t}$ represent the weights, and ${\mathbf {b}}_{t}$ represents the bias. Then, we use a fully connected layer as the readout layer to obtain the drug representation. By applying this module to the whole dataset, we obtain the sequence-based feature matrix ${\mathbf {S}} \in {\mathbb {R}}^{n \times d_{g}}$.

Note that we should input a fix-sized matrix into the BiGRU layer. However, the length of the SMILES sequence varies. We use the approximately average length of the sequences in the dataset as the fixed length and apply zero-padding and cutting operations.

### Multi-type feature fusion module

**Fig. 4:**
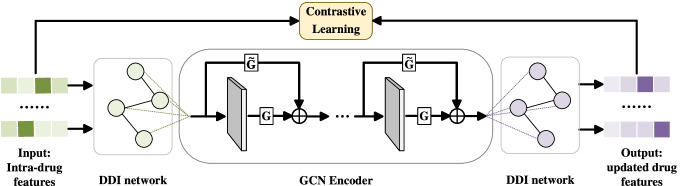
Overview of MFFM, where ${\mathbf {G}}$ is gating and $\tilde{{\mathbf {G}}}$ is 1-gating. The MFFM takes the intra-drug features as the initial node features in DDI network, and then update the node representation by multi-layer graph convolution neural network with gating

We combine the feature matrices ${\mathbf {G}}$ and ${\mathbf {S}}$ obtained above to obtain the intra-drug features, namely ${\mathbf {H}}={\mathbf {G}}\bigoplus {\mathbf {S}}$. In order to fuse the intra-drug features with the external DDI features, we design a GCN encoder with the gating mechanism. Specifically, we take the intra-drug features as the initial node features in the interaction graphs, and then update the node representation by multi-layer GCN. The Multi-type Feature Fusion Module (MFFM) is shown in Fig. 4.

For drug $d_{i}$, the output of $r^{t h}$ layer is as follows:

18 $$\begin{aligned} {\mathbf {z}}_{i}^{r}={\text {ReLU}}(\sum _{j \in {\mathcal {N}}(i)}\tilde{{\mathbf {A}}}_{ij} {\mathbf {z}}_{j}^{r-1} {\mathbf {W}}_{u}^{r} ) \end{aligned}$$

where ${\mathbf {W}}_{u}^{r}$ is learnable weight parameter. $\tilde{{\mathbf {A}}}_{ij}$ is the component of the normalized adjacency matrix $\tilde{{\mathbf {A}}}$. $\tilde{{\mathbf {A}}}=\hat{{\mathbf {K}}}^{-\frac{1}{2}}\left( {\mathbf {A}}+{\mathbf {I}}_{n}\right) \hat{{\mathbf {K}}}^{-\frac{1}{2}}$ where $\hat{{\mathbf {K}}}_{\mathbf {i i}}=\sum _{\mathrm {j}}\left( {\mathbf {A}}+{\mathbf {I}}_{n}\right) _{\mathrm {i j}}$. We can add multiple GCN layers to expand the neighborhood of label propagation, but it may also cause the increase of noisy information. Meanwhile, the neighborhoods of different orders contain different information. Therefore, we utilize the gating mechanism [37] to control how much neighborhood information is passed to the node. The process is as follows:

19 $$\begin{aligned} T({\mathbf {z}}_{i}^{r-1})&=\sigma ({\mathbf {W}}^{r-1} {\mathbf {z}}_{i}^{r-1}+{\mathbf {b}}^{r-1}) \end{aligned}$$

20 $$\begin{aligned} {\mathbf {z}}_{i}^{r}&={\mathbf {z}}_{i}^{r} \odot T({\mathbf {z}}_{i}^{r-1})+{\mathbf {z}}_{i}^{r-1} \odot (1-T({\mathbf {z}}_{i}^{r-1})) \end{aligned}$$

where $T({\mathbf {c}}^{r-1})$ represents the gating weight of the $(r-1)^{t h}$ layer, $({\mathbf {W}}^{r-1}, {\mathbf {b}}^{r-1})$ are weight matrix and bias variable of the $(r-1)^{t h}$ layer. After multi-layer GCN, we finally obtain the feature matrix ${\mathbf {Z}} \in {\mathbb {R}}^{n \times d_{g}}$ for drugs in DDI Network.

In addition, inspired by MIRACLE, the module uses the graph contrastive learning approach to balance the information inside and outside of the drug. For the drug $d_{i}$, we take itself and its first-order neighboring nodes as positive samples *P* and the nodes not in first-order neighbors as negative samples *N*. We design a learning objective, which made external features of drug $d_{i}$ consistent with internal features of positive samples and distinct from internal features of negative samples, defined as follows:

21 $$\begin{aligned} {\mathscr {L}}_{c}=-\log \sigma (f_{\mathrm {D}}({\mathbf {h}}_{i}, {\mathbf {z}}_{i}))-\log \sigma (1\!-\!f_{\mathrm {D}}(\tilde{{\mathbf {h}}}_{i}, {\mathbf {z}}_{i})) \end{aligned}$$

where $f_{\mathrm {D}}(\cdot )\!: \!{\mathbb {R}}^{d_{g}} \!\times \!{\mathbb {R}}^{d_{g}} \!\longmapsto \!{\mathbb {R}}$ is the discriminator function, which scores agreement between the two vectors of the input. Here we set it to the point product operation.

### DDI prediction

Firstly, we obtain an interaction link representation by multiplying two drug representation. Then, we input it into the MLP to get the prediction score:

22 $$\begin{aligned} {\hat{y}}_{i j}=\sigma \left( {\text {MLP}}\left( z_{i} \odot z_{j}\right) \right) \end{aligned}$$

where MLP consists of two fully connected layers.

Our learning objective is to minimize the distance between the predictions and the true labels. The specific formula is as follows:

23 $$\begin{aligned} {\mathscr {L}}_{r}= -\sum _{l_{i j} \in {\mathcal {L}}} y_{i j} \log ({\hat{y}}_{i j})+(1-y_{i j}) \log (1-{\hat{y}}_{i j}) \end{aligned}$$

where $y_{i j}$ is the real label for drug pair $(d_{i},d_{j})$. Then, we unify the DDI prediction task and the contrastive learning task into a learning framework. Formally, the learning objective of our model is:

24 $$\begin{aligned} {\mathscr {L}}={\mathscr {L}}_{r}+\alpha {\mathscr {L}}_{c} \end{aligned}$$

where $\alpha$ is a hyper-parameter used to control the magnitude of contrastive task.

## Results

In this section, we design various experiments to demonstrate the superiority of the model MFFGNN.

### Experimental setup

**Datasets. ** To verify the validity of our model on datasets with different scales, we evaluate the proposed model in small, medium, and large datasets. In the small-scale dataset, the number of drugs is relatively small, but fingerprints of all drugs are available. In the medium-scale dataset, although the number of drugs is relatively large, there is only the same number of labeled DDI links as in small-scale dataset. In the large-scale dataset, most of drugs lack many fingerprints. Detailed information about the datasets can be seen in Table 1.

**Table 1:** Detailed information about the datasets

Dataset	Drugs	DDI links	Information
ZhangDDI [46]	548	48,548	Similarity
ChCh-Miner [47]	1514	48,514	–
DeepDDI [30]	1861	192,284	Polypharmacy side-effect

Note that we removed the SMILES sequences that cannot construct the graph in the dataset.

*Baselines* To demonstrate the superiority of our model, we compare MFFGNN with the following state-of-the-art models:

- *SSP-MLP* [30]: This approach used the names and structural information of drug-drug or drug-food pairs as inputs and applied Structural Similarity Profile (SSP) and MLP for classification. We name this model as SSP-MLP.
- *Multi-Feature Ensemble* [46]: This approach combined multiple types of data and proposed a collective framework. We name this model as Ens.
- *GCN* [48]: This approach applied GCN to perform semi-supervised node classification. We use GCN to extract structural information of drugs for DDI prediction.
- *GAT* [35]: This approach used GAT to perform node classification task. We apply GAT to extract drug features in interaction graph for DDI prediction.
- *SEAL-C/AI* [49]: This approach performs semi-supervised graph classification tasks from a hierarchical graph perspective. We apply this model to obtain drug features for DDI prediction.
- *NFP-GCN* [39]: This approach designs a GCN for learning molecular fingerprints. We name this model as NFP-GCN.
- *MIRACLE* [42]: This approach simultaneously learned the inter-view molecular structure information and intra-view interaction information of drugs for DDI prediction.
- *MFs* [50]: This approach only used molecular fingerprints as input to the DDI network to predict DDIs, we name this model as MFs.
- We also consider several multi-type DDI prediction methods and apply them to binary classification tasks, i.e. DPDDI [14], SSI-DDI [18], DDIMDL [7], MUFFIN [20].

*Implementation details* For the division of the datasets, the splitting method is the same as MIRACLE [42]. We divide 80% of each dataset into the training set, 20% into the test set, and 20% of the training set are randomly sampled as the validation set. The dataset only contains positive drug pairs. For negative training samples, we select the same number of negative drug pairs [51].

We utilize Adam [52] optimizer to train the model and Xavier [53] initialization to initialize the model. We utilize the exponential decay method to set the learning rate, where the initial learning rate is 0.0001 and the multiplication factor is 0.96. The model applies a dropout [54] layer to the output of each intermediate layer, where the dropout rate is 0.3. We set the dimension of the atom-level and drug-level representations as 256. We set $K=2$ in the multi-head attention mechanism. To evaluate the effectiveness of the model MFFGNN, we consider three metrics, including Area Under the Receiver Operating Characteristic curve (AUROC), Area Under the Precision-recall Curve (AUPRC) and F1.

### Comparison results

To verify the validity of the proposed MFFGNN, we compare MFFGNN with state-of-the-art models for DDI prediction on three datasets with different scales. Over ten repeated experiments, we give the mean and standard deviation. The best results are highlighted in bold.

*Comparison on the ZhangDDI dataset* We compare the MFFGNN model with state-of-the-art models on the ZhangDDI dataset, and the results are shown in Table 2. The results of these baselines are obtained from Table 2 in Ref. [42]. As can be seen, the methods considering multiple features, such as Ens, SEAL-C/AI, NFP-GCN and MIRACLE, perform better than the methods considering only one feature. However, the MFFGNN has the best performance. MFFGNN considers not only the topological structure information in molecular graphs and the interaction information between drugs, but also the local chemical context in SMILES sequences. This indicates that multi-type feature fusion can improve the performance of the model.

**Table 2:** Comparison results on ZhangDDI dataset

Method	AUROC	AUPRC	F1
SSP-MLP	92.51 ± 0.15	88.51 ± 0.66	80.69 ± 0.81
Ens	95.20 ± 0.14	92.51 ± 0.15	85.41 ± 0.16
GCN	91.91 ± 0.62	88.73 ± 0.84	81.61 ± 0.39
GAT	91.49 ± 0.29	90.69 ± 0.10	80.93 ± 0.25
SEAL-C/AI	92.93 ± 0.19	92.82 ± 0.17	84.74 ± 0.17
NFP-GCN	93.22 ± 0.09	93.07 ± 0.46	85.29 ± 0.17
MIRACLE	98.95 ± 0.15	98.17 ± 0.06	93.20 ± 0.27
MFFGNN	**99.06 ± 0.08**	**98.83 ± 0.16**	**97.97 ± 0.25**

**Table 3:** Comparison results on ChCh-Miner dataset

Method	AUROC	AUPRC	F1
GCN	82.84 ± 0.61	84.27 ± 0.66	70.54 ± 0.87
GAT	85.84 ± 0.23	88.14 ± 0.25	76.51 ± 0.38
SEAL-C/AI	90.93 ± 0.19	89.38 ± 0.39	84.74 ± 0.48
NFP-GCN	92.12 ± 0.09	93.07 ± 0.69	85.41 ± 0.18
MIRACLE	96.15 ± 0.29	95.57 ± 0.19	92.26 ± 0.09
MFFGNN	**97.02 ± 0.25**	**98.45 ± 0.06**	**96.94 ± 0.39**

**Fig. 5:**
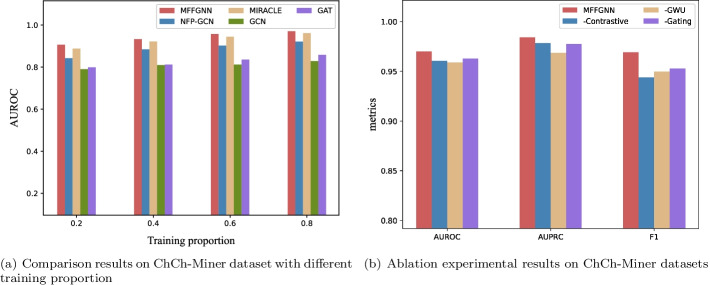
Experimental results on ChCh-Miner dataset

*Comparison on the ChCh-Miner dataset* Because the ChCh-Miner dataset lacks fingerprints and side-effect information, we only compare the MFFGNN with the graph-based models, and the results are shown in Table 3. The results of these baselines are obtained from Table 3 in Ref. [42]. As shown in Table 3, MFFGNN outperforms all baselines in all metrics, indicating that MFFGNN still maintain its effectiveness on the dataset with few labeled data. In addition, we obtain labeled training data with different amounts by adjusting the proportion of the training set on the ChCh-Miner dataset. This can analyze the robustness of the MFFGNN. We compare MFFGNN with other methods, and the results are shown in Fig. 5a. The results show that MFFGNN has high performance even in a small amount of labeled data. The reason could be that (i) our model fuses topological structure, local chemical context and DDI relationships; (ii) our model extracts both the global features for the molecular graph and the local features for the atoms of the molecular graph; (iii) our model sets a gating mechanism for each graph convolution layer to prevent over-smoothing when stacking multi-layer GCN.

**Table 4:** Comparison results on DeepDDI dataset

Method	AUROC	AUPRC	F1
SSP-MLP	92.28 ± 0.18	90.27 ± 0.28	79.71 ± 0.16
GCN	85.53 ± 0.17	83.27 ± 0.31	72.18 ± 0.22
GAT	84.84 ± 0.23	81.14 ± 0.25	73.51 ± 0.38
SEAL-C/AI	92.83 ± 0.19	90.44 ± 0.39	80.70 ± 0.48
MFs	91.54 ± 0.04	89.82 ± 0.24	83.05 ± 0.5
DPDDI	92.79 ± 0.38	91.15 ± 0.52	85.54 ± 0.40
SSI-DDI	**96.14 ± 0.06**	94.63 ± 0.47	92.27 ± 0.14
DDIMDL	94.85 ± 0.71	93.48 ± 0.07	82.31 ± 0.44
MUFFIN	95.26 ± 0.12	94.47 ± 0.28	91.22 ± 0.48
MIRACLE	95.51 ± 0.27	92.34 ± 0.17	83.60 ± 0.33
MFFGNN	95.39 ± 0.25	**96.81 ± 0.16**	**92.54 ± 0.61**

The best results are highlighted in bold

*Comparison on the DeepDDI dataset* To verify the scalability of MFFGNN, we perform comparative experiments on the DeepDDI dataset, and the results are shown in Table 4. Because there may be missing information in the large-scale dataset, we only choose the SSP-MLP model. And the NFP-GCN model has worse performance and space limitation. We also ignore the experimental results. We use 881 dimensional molecular fingerprints as the initial node features in the DDI graph for DDIs prediction. Meanwhile, we degrade multi-type DDI prediction methods and obtain binary prediction results on DeepDDI dataset.

As shown in Table 4, MFFGNN has high AUROC, AUPRC and F1. The MFs model is relatively poor in all metrics, which only contains one drug feature. Single feature can not comprehensively represent drug information, which will ultimately affect the prediction results. However, MFFGNN integrates the features from drug sequences and molecular graphs to input into DDI graph, so that a more comprehensive drug information can be learned. Although the SSI-DDI and MIRACLE models have higher AUROC metric than MFFGNN, MFFGNN has the highest AUPRC and F1 values. In general, the AUPRC metric is more important than the AUROC metric, because it penalizes false positive DDIs better. F1 focuses on the proportion that can correctly predict DDIs. The imbalance of the data in the DeepDDI dataset may have a negative impact on the AUROC metrics of our model. However, this does not affect the performance of MFFGNN.

**Fig. 6:**
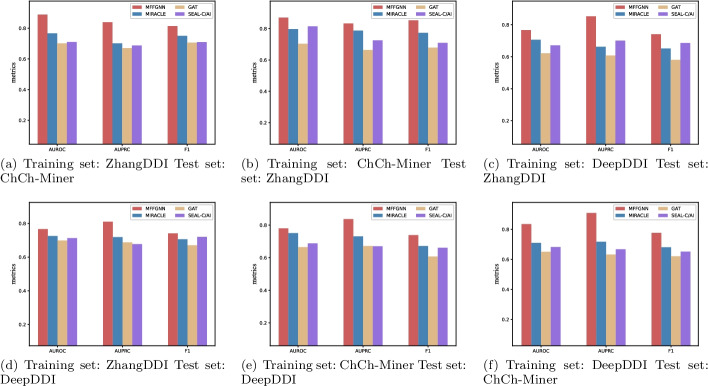
Cross-dataset experimental results

*Cross-dataset evaluations* To further evaluate that MFFGNN has good generalization performance, we perform cross-dataset evaluations. One dataset serves as the training set, while the other two serve as test sets. Because of the poor performance of other methods, we compare MFFGNN to three methods, including GAT, SEAL-C/AI and MIRACLE, and the results are shown in Fig. 6. As shown in figures, MFFGNN outperforms the other methods in AUROC, AUPRC and F1. From the above results, it can be shown that our model can predict drug-drug interaction with steady accuracy, independent of the scale of the datasets. Through this experiment, we can also verify that MFFGNN has good generalization performance.

### Ablation study

**Table 5:** The performance of different types of features on ChCh-Miner dataset

Method	AUROC	AUPRC	F1
S	90.17 ± 0.04	90.27 ± 0.18	89.14 ± 0.08
M	92.87 ± 0.74	92.55 ± 0.40	90.93 ± 0.56
I	93.23 ± 0.01	92.74 ± 0.15	90.28 ± 0.31
S+I	96.01 ± 0.83	96.89 ± 0.76	94.99 ± 0.23
S+M	95.49 ± 0.72	95.33 ± 0.54	95.02 ± 0.16
M+I	96.25 ± 0.05	97.23 ± 0.02	94.87 ± 0.05
S+M+I	**97.02 ± 0.25**	**98.45 ± 0.06**	**96.94 ± 0.39**

The best results are highlighted in bold

* S* SMILES sequence, *M* molecular graph, *I* interaction

**Table 6:** Ablation experimental results on ChCh-Miner dataset

Method	AUROC	AUPRC	F1
–GWU	95.89 ± 0.15	97.26 ± 0.18	94.97 ± 0.67
–Gating	96.28 ± 0.23	97.78 ± 0.31	95.28 ± 0.20
–Contrastive	96.07 ± 0.28	97.85 ± 0.15	94.38 ± 0.06
MFFGNN	**97.02 ± 0.25**	**98.45 ± 0.06**	**96.94 ± 0.39**

The best results are highlighted in bold

In order to verify the validity of each type of feature of drugs, we carry out DDI predictions using each type of feature or combination of feature on ChCh-Miner datasets. The experimental results are shown in Table 5. The best results are highlighted in bold.

As shown in Table 5, deleting any one of these three types of the features will damage performance. The performance is best when the three types of features are considered simultaneously. In addition, among single feature, considering only the interaction information between drugs or the topological information of the molecular graph, the model has the great performance. Among pairwise feature combinations, considering the interaction information between drugs and the topological information of the molecular graph simultaneously performs best, and pairwise feature combinations can significantly improve performance than single feature. This suggests that multi-feature integration can better represent drugs and improve prediction results.

Our model considers the global features for the molecular graph and the local features for the atoms of the molecular graph. In order to study its effectiveness, we design a variant, namely -GWU. -GWU ignores the global information in molecular graphs. As shown in Table 6, deleting the global features will damage performance. To study the validity of contrastive learning, we design a variant, called -Contrastive. This variant removes the contrastive learning from the framework. As shown in Table 6, -Contrastive is inferior to MFFGNN in all metrics. The results show that contrastive learning is beneficial to assist drug feature learning.

MFFGNN contains a GCN encoder with the gating mechanism to fully utilize the neighborhood information of different order. In order to study its effectiveness, we conduct a comparative experiment based on whether there is gating or not, and the results are shown in Table 6. The performance of the model without gating is lower than that of the model with gating. It can be proved that GCN encoder with gating is beneficial to predict DDI. From Fig. 5b, we can intuitively see the effectiveness of each component of the proposed MFFGNN.

### Parameter analysis

In this section, we analyze several key parameters in the model by performing experiments on the ZhangDDI dataset, including $\alpha$ in the objective function of our model, the dimensionality of drug representation $d_{g}$, sequence length $L_{s}$, learning rate $l_{r}$, the number of GCN layers $L_{m}$ and *k* of the k-head attention in the MGFEM module. We study the influence of different key parameters settings on MFFGNN by fixing other parameters.

**Fig. 7:**
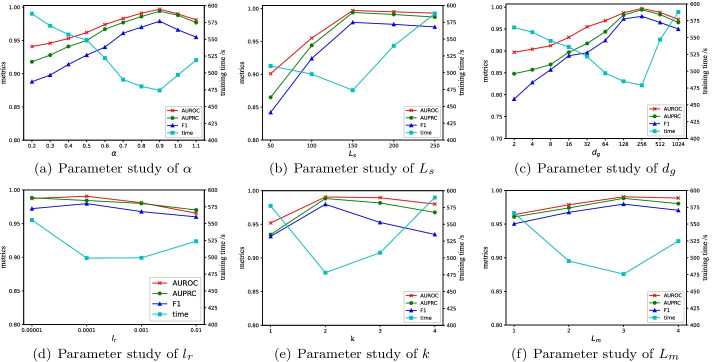
Parameter study on ZhangDDI dataset

In order to study the optimal setting of $\alpha$ in the objective function of our model, we vary $\alpha$ from 0.1 to 1.0 and fix other parameters, the results are shown in Fig. 7a. We observe that the three metrics are optimal when $\alpha$ is set to 0.9. On the whole, the non-zero nature of $\alpha$ proves the importance of contrastive learning in the model.

When training the BiGRU, we need to input a fix-sized matrix. However, the length of SMILES sequences varies. Therefore, we fix the length of the input sequence at some value and apply zero-padding and cutting operations. To study the optimal setting of sequence length, we vary $L_{s}$ from 50 to 250 and fix other parameters, the results are shown in Fig. 7b. Because most of the SMILES sequences in the dataset are less than 150 and greater than 100, the model performance is optimal when $L_{s}=150$. When $L_{s}=150$, most of the sequences do not need to be cut, and little information is lost. But, when $L_{s}=100$, most of the sequences will lose information, and the performance is low. When the sequence length is greater than 150, even if zero-paddings are applied, the performance degradation could be trivial, because it contains enough sequence information.

In order to study the optimal setting of $d_{g}$, we change it from 2 to 1024 and fix other parameters, and the results are shown in Fig. 7c. When $d_{g}$ is set to 256, the three metrics are optimal, and the model achieves the best performance. Specifically, with the increase of the dimensionality of drug representation, MFFGNN can extract more useful information. However, a too high dimensionality may increase noise and lead to performance degradation. Similarly, in order to study the optimal setting of $l_{r}$, we change $l_{r}$ with $\{0.01,0.001,0.0001,0.00001\}$ and fix other parameters, the results are shown in Fig. 7d. When $l_{r}$ = 0.0001, the model performance is best.

In order to study the optimal setting of $L_{m}$ and *k* of the k-head attention in the MGFEM module, we change it from 1 to 4 and fix other parameters, the results are shown in Fig. 7e, f. For *k* of k-head attention, when $k=2$, the model performance is the best. As seen from the figure, as the $L_{m}$ increases, the MFFGNN performance improves. When $L_{m}=3$, the three metrics are optimal and the model achieves the best performance. However, too many layers may cause overfitting and lead to performance degradation.

## Discussions

Drug-Drug prediction has always been a worthy research direction in pharmacology. Most of the existing methods for predicting drug-drug interactions only consider single drug feature. However, single drug feature cannot comprehensively represent drug information, which will ultimately affect the prediction results. Our proposed model takes into account not only the topological structure information in molecular graphs and the interaction information between drugs, but also the local chemical context in SMILES sequences. Multiple drug features will represent the drug information more comprehensively. We perform DDI predictions using each type of feature or combination of features, and the experimenta results are shown in Table 5. When the three types of features are considered simultaneously, the model has the best performance.

When extracting information from the molecular graph, we extract the local feature of the atoms and the global feature of the whole molecular graph. This facilitates the remote propagation of the information in graph. We demonstrate the importance of the global features of the molecular graphs in the ablation experiments, and the results are given in Table 6. In addition, To verify evaluate that MFFGNN has good generalization performance, we perform cross-dataset evaluations, and the results are given in Fig. 6. As shown in figures, our model can predict drug-drug interaction with stable accuracy, regardless of the scale of the dataset. However, our model also has some limitations, for example, it does not extend to multi-type DDI prediction tasks. In future work, we will further generalize the model to predict multi-type DDIs events.

## Conclusions

In this paper, we propose a novel end-to-end learning framework for DDI prediction, namely MFFGNN, which can efficiently fuse the information from drug molecular graphs, SMILES sequences and DDI graphs. The MFFGNN model utilizes the molecular graph feature extraction module to extract global and local features in molecular graphs. Moreover, in the multi-type feature fusion module, we set up the gating mechanism to control how much neighborhood information is passed to the node. We perform extensive experiments on multiple real datasets. The results show that the MFFGNN model consistently outperforms other state-of-the-art models.

## Data Availability

The datasets generated and/or analysed during the current study are available in the Github repository, https://github.com/kaola111/mff

## Abbreviations

MFFGNN: Multi-type Feature Fusion based on Graph Neural Network
DDIs: Drug-Drug interactions
SMILES: Simplified Molecular Input Line Entry System
GNN: Graph neural network
GCN: Graph convolution network
MLP: Multi-layer perceptro
SSFEM: SMILES sequence feature extraction module
MGFEM: Molecular graph feature extraction module
MFFM: Multi-type feature fusion module
GWU: Graph warp unit
BiGRU: Bi-directional gate recurrent unit

## References

[1] Zhang W, Jing K, Huang F, Chen Y, Li B, Li J, Gong J (2019). Sflln: a sparse feature learning ensemble method with linear neighborhood regularization for predicting drug-drug interactions. Inf Sci.

[2] Yan C, Duan G, Zhang Y, Wu FX, Pan Y, Wang J (2020). Predicting drug-drug interactions based on integrated similarity and semi-supervised learning. IEEE/ACM Trans Comput Biol Bioinform.

[3] Zhang Y, Qiu Y, Cui Y, Liu S, Zhang W (2020). Predicting drug-drug interactions using multi-modal deep auto-encoders based network embedding and positive-unlabeled learning. Methods.

[4] Zhu J, Liu Y, Zhang Y, Li D (2020). Attribute supervised probabilistic dependent matrix tri-factorization model for the prediction of adverse drug-drug interaction. IEEE J Biomed Health Inf.

[5] Qiu Y, Zhang Y, Deng Y, Liu S, Zhang W (2021). A comprehensive review of computational methods for drug-drug interaction detection. IEEE/ACM Trans Comput Biol Bioinform.

[6] Deng Y, Qiu Y, Xu X, Liu S, Zhang Z, Zhu S, Zhang W (2022). Meta-ddie: predicting drug-drug interaction events with few-shot learning. Brief Bioinform.

[7] Deng Y, Xu X, Qiu Y, Xia J, Zhang W, Liu S (2020). A multimodal deep learning framework for predicting drug-drug interaction events. Bioinformatics.

[8] Zhang W, Chen Y, Li D, Yue X (2018). Manifold regularized matrix factorization for drug-drug interaction prediction. J Biomed Inform.

[9] Huang K, Xiao C, Hoang T, Glass L, Sun J. Caster: Predicting drug interactions with chemical substructure representation. In: Proceedings of the AAAI Conference on Artificial Intelligence. 2020;34:702–9.

[10] Li P, Wang J, Qiao Y, Chen H, Yu Y, Yao X, Gao P, Xie G, Song S (2021). An effective self-supervised framework for learning expressive molecular global representations to drug discovery. Brief Bioinform.

[11] Wang F, Lei X, Liao B, Wu F-X (2022). Predicting drug-drug interactions by graph convolutional network with multi-kernel. Brief Bioinform.

[12] Feeney A et al. Relation matters in sampling: A scalable multi-relational graph neural network for drug-drug interaction prediction. arXiv preprint arXiv:2105.13975 2021.

[13] Purkayastha S, Mondal I, Sarkar S, Goyal P, Pillai JK. Drug-drug interactions prediction based on drug embedding and graph auto-encoder. In: 2019 IEEE 19th International Conference on Bioinformatics and Bioengineering (BIBE), 2019;547–552 . IEEE

[14] Feng Y-H, Zhang S-W, Shi J-Y (2020). Dpddi: a deep predictor for drug-drug interactions. BMC Bioinform.

[15] Dai Y, Guo C, Guo W, Eickhoff C (2021). Drug-drug interaction prediction with wasserstein adversarial autoencoder-based knowledge graph embeddings. Brief Bioinform.

[16] Lyu T, Gao J, Tian L, Li Z, Zhang P, Zhang J (2019). Mdnn: a multimodal deep neural network for predicting drug-drug interaction events. Science.

[17] Yu Y, Huang K, Zhang C, Glass LM, Sun J, Xiao C (2021). Sumgnn: multi-typed drug interaction prediction via efficient knowledge graph summarization. Bioinformatics.

[18] Nyamabo AK, Yu H, Shi J-Y (2021). Ssi-ddi: substructure-substructure interactions for drug-drug interaction prediction. Brief Bioinform.

[19] Nyamabo AK, Yu H, Liu Z, Shi J-Y (2022). Drug-drug interaction prediction with learnable size-adaptive molecular substructures. Brief Bioinform.

[20] Chen Y, Ma T, Yang X, Wang J, Song B, Zeng X (2021). Muffin: multi-scale feature fusion for drug-drug interaction prediction. Bioinformatics..

[21] Bahdanau D et al. Neural machine translation by jointly learning to align and translate. arXiv preprint arXiv:1409.0473 2014.

[22] Battaglia PW, Pascanu R, Lai M, Rezende D, Kavukcuoglu K (2016). Interaction networks for learning about objects, relations and physics. Science..

[23] Ishiguro K, Maeda Si, Koyama M. Graph warp module: an auxiliary module for boosting the power of graph neural networks in molecular graph analysis. arXiv preprint arXiv:1902.01020 2019.

[24] Duke JD, et al. Literature based drug interaction prediction with clinical assessment using electronic medical records: novel myopathy associated drug interactions 2012. 10.1371/journal.pcbi.1002614 PMC3415435 22912565

[25] Takeda T, Hao M, Cheng T, Bryant SH, Wang Y (2017). Predicting drug-drug interactions through drug structural similarities and interaction networks incorporating pharmacokinetics and pharmacodynamics knowledge. J Cheminform.

[26] Vilar S, Uriarte E, Santana L, Lorberbaum T, Hripcsak G, Friedman C, Tatonetti NP (2014). Similarity-based modeling in large-scale prediction of drug-drug interactions. Nat Protoc.

[27] Fokoue A, Sadoghi M, Hassanzadeh O, Zhang P. Predicting drug-drug interactions through large-scale similarity-based link prediction. In: European Semantic Web Conference, 2016;774–789 . Springer

[28] Ma T, Xiao C, Zhou J, Wang F. Drug similarity integration through attentive multi-view graph auto-encoders. arXiv preprint arXiv:1804.10850 2018.

[29] Kastrin A, Ferk P, Leskošek B (2018). Predicting potential drug-drug interactions on topological and semantic similarity features using statistical learning. PLoS ONE.

[30] Ryu JY (2018). Deep learning improves prediction of drug-drug and drug-food interactions. Proc Natl Acad Sci.

[31] Xu N et al. Mr-gnn: Multi-resolution and dual graph neural network for predicting structured entity interactions. arXiv preprint arXiv:1905.09558 2019.

[32] Ma T et al. Genn: predicting correlated drug-drug interactions with graph energy neural networks. arXiv preprint arXiv:1910.02107 2019.

[33] Shang J, Xiao C, Ma T, Li H, Sun J. Gamenet: Graph augmented memory networks for recommending medication combination 2018.

[34] Hamilton, W.L., Ying, R., Leskovec, J.: Inductive representation learning on large graphs. In: Proceedings of the 31st International Conference on Neural Information Processing Systems, 2017;1025–1035

[35] Veličković P et al. Graph attention networks. arXiv preprint arXiv:1710.10903 2017.

[36] Schlichtkrull M, Kipf TN, Bloem P, Van Den Berg R, Titov I, Welling M. Modeling relational data with graph convolutional networks. In: European Semantic Web Conference, 2018;593–607 . Springer

[37] Rahimi A et al. Semi-supervised user geolocation via graph convolutional networks. arXiv preprint arXiv:1804.08049 2018.

[38] Zitnik M (2018). Modeling polypharmacy side effects with graph convolutional networks. Bioinformatics.

[39] Duvenaud D et al. Convolutional networks on graphs for learning molecular fingerprints. arXiv preprint arXiv:1509.09292 2015.

[40] Lin, X., et al.: Kgnn: Knowledge graph neural network for drug-drug interaction prediction. In: IJCAI, 2020;380:2739–2745.

[41] Bai Y et al. Bi-level graph neural networks for drug-drug interaction prediction. arXiv preprint arXiv:2006.14002 2020.

[42] Wang Y et al. Multi-view graph contrastive representation learning for drug-drug interaction prediction. In: Proceedings of the Web Conference 2021, 2021;2921–2933.

[43] Landrum G (2013). RDKit: a software suite for cheminformatics, computational chemistry, and predictive modeling.

[44] Chung J, Gulcehre C, Cho K, Bengio Y. Empirical evaluation of gated recurrent neural networks on sequence modeling. arXiv preprint arXiv:1412.3555 2014.

[45] Quan Z et al. A system for learning atoms based on long short-term memory recurrent neural networks. In: 2018 IEEE International Conference on Bioinformatics and Biomedicine (BIBM), 2018;728–733. IEEE

[46] Zhang W (2017). Predicting potential drug-drug interactions by integrating chemical, biological, phenotypic and network data. BMC Bioinformatics.

[47] Marinka Zitnik SM, Rok Sosič, Leskovec J. BioSNAP Datasets: Stanford Biomedical Network Dataset Collection. http://snap.stanford.edu/biodata 2018

[48] Kipf TN, Welling M. Semi-supervised classification with graph convolutional networks. arXiv preprint arXiv:1609.02907 2016

[49] Li J et al. Semi-supervised graph classification: A hierarchical graph perspective. In: The World Wide Web Conference, 2019;972–982

[50] Kim S, Chen J, Cheng T, Gindulyte A, He J, He S, Li Q, Shoemaker BA, Thiessen PA, Yu B (2019). Pubchem 2019 update: improved access to chemical data. Nucleic Acids Res.

[51] Chen X, Liu X, Wu J. Drug-drug interaction prediction with graph representation learning. In: 2019 IEEE International Conference on Bioinformatics and Biomedicine (BIBM), 2019;354–361. IEEE

[52] Kingma DP, Ba J. Adam: A method for stochastic optimization. arXiv preprint arXiv:1412.6980 2014

[53] Glorot X, Bengio Y. Understanding the difficulty of training deep feedforward neural networks. In: Proceedings of the Thirteenth International Conference on Artificial Intelligence and Statistics, 2010;249–256. JMLR Workshop and Conference Proceedings

[54] Srivastava N (2014). Dropout: a simple way to prevent neural networks from overfitting. J Mach Learn Res.

